# Responding to health policy recommendations on managing opioid use disorder during Russia's invasion of Ukraine: Divergent responses from the frontline to the west

**DOI:** 10.3389/fpubh.2022.1044677

**Published:** 2023-01-13

**Authors:** Roman Ivasiy, Samy J. Galvez de Leon, Anna Meteliuk, Tetiana Fomenko, Iryna Pykalo, Daniel J. Bromberg, Lynn M. Madden, Scott O. Farnum, Zahedul Islam, Frederick L. Altice

**Affiliations:** ^1^Section of Infectious Diseases, Yale School of Medicine, New Haven, CT, United States; ^2^International Charitable Foundation Alliance for Public Health, Kyiv, Ukraine; ^3^Ukrainian Institute of Public Health Policy, Kyiv, Ukraine; ^4^Center for Interdisciplinary Research on AIDS, Yale University, New Haven, CT, United States; ^5^Department of Social and Behavioral Sciences, Yale School of Public Health, Yale University, New Haven, CT, United States; ^6^APT Foundation, New Haven, CT, United States; ^7^Division of Epidemiology of Microbial Diseases, Yale School of Public Health, New Haven, CT, United States

**Keywords:** opioid use disorder, Ukraine, Russian-Ukrainian war, methadone (MTD), war and migration, internally displaced people (IDPs), opioid agonist therapies

## Abstract

**Summary:** Russia's invasion of Ukraine on February 24, 2022, followed by Ukraine's Martial law, has disrupted the routine delivery of healthcare services, including opioid agonist treatment (OAT) programs. Directors (chief addiction treatment physicians) of these programs in each region had flexibility with implementing a series of adaptations to their practice to respond to war disruptions like mass internal displacement and legislation updates allowing more flexibility with OAT distribution policies and take-home dosing regulations. We conducted 8 in-depth interviews with directors from seven regions of Ukraine to describe their experiences providing OAT during a specific time during the war and the local crisis-response approach under the emergency policy updates. We categorized their experiences according to the level of exposure to conflict in each region and displacement of patients across the country, which may provide future guidance for OAT provision during the conflict.

## Introduction

International authorities and scholars call for and are committed to ensuring continued medical treatment for people in Ukraine as the unprovoked war inflicted by Russia evolves (1–3). On February 24, 2022, the Russian Federation launched a full-scale war on and invaded Ukraine. Ukraine has Europe's second-worst HIV epidemic (second to Russia) (4), fueled by transmission among people who inject drugs (PWID) and their sexual partners (5), with opioids being the most prevalent drug injected among PWID (6). With an estimated 346,000 PWID and 360,000 people with HIV (PWH), these dual epidemics required innovative and linked strategies for effective control. Scaling up opioid agonist therapies (OAT) with methadone or buprenorphine is among the most effective and cost-effective strategies for controlling Ukraine's HIV epidemic (7–11). Over the past decade, scale-up has been guided by effective implementation strategies using the Network for the Improvement of Addiction Treatment (NIATx) (12), including since Russia's first invasion in 2014. It has been used to guide the modification of policies for prescribing OAT in 2016 and guide public health responses during the COVID-19 pandemic (13). Yet, a series of implementation disruptors have greatly impacted health policies that impact OAT scale-up, including the illegal occupation of Crimea (March 18^th^, 2014), military conflict in partially occupied areas of Donetsk and Luhansk regions (since April 6^th^, 2014), substantial changes to Order 200 (the regulatory guidance that has governed OAT delivery since 2012) in 2016 and 2020, the COVID-19 pandemic (2020), and introduction of the national healthcare system reform (2020), which provided supplemental resources for clinicians. These disruptors prepared Ukraine, in part, to respond rapidly to the new invasion (1, 12, 14). As the war has now disrupted every aspect of life in Ukraine, OAT scale-up and sustainment remain threatened, posing a significant challenge to managing the intertwined HIV and opioid use disorder (OUD) epidemics in Ukraine.

Narcologists, subspecialists in addiction psychiatry, and physicians responsible for OAT delivery across the country learned first-hand the consequences of military conflict in Crimea and parts of Eastern Ukraine. The chief narcologist in each region is responsible for overseeing OAT procurement and scale-up and has broad latitude within the regulatory framework in deciding implementation responses and actions related to policy changes. The regulatory framework of Order 200 was especially rigid until November 2016 when it allowed some patients to transition from daily supervision to up to 10 days of take-home dosing (THD) after 6 months of documented sobriety. This 2016 change also removed restrictions that patients fail two detoxification attempts and allowed OAT to be delivered outside specialized treatment clinics (15), including in prisons and primary care clinics (16, 17). Order 200 did not, however, remove requirements for official registration as a drug user and remove restrictions for driving and some types of employment (15). Not all regions, however, adopted policy changes immediately, resulting in substantial regional differences both in terms of coverage and THD. Allowing take-home dosing, however, was especially controversial as it was new in Ukraine and clinicians were especially concerned about overdose. Guidance during the COVID-19 pandemic, however, further disrupted OAT scale-up by allowing narcologists to rapidly increase the proportion of patients receiving THD from 53% to 82% within 30 days (13). Similarly, there were regional differences in the adoption of this policy as the chief narcologist for each region had different levels of concern about THD, yet considerable latitude in implementing these recommended changes (13).

In the 6 weeks before the widespread bombing that started in Ukraine on February 24, 2022, the government and non-governmental sectors began to plan for a potential invasion, with most expecting a limited invasion or none at all (12). Instead, there was a massive offensive by Russia with an invasion from the north, south, and east of the country, resulting in the major internal displacement of persons along with the largest refugee crisis in Europe since World War II (3). The Ukrainian government emergently introduced updated guidance for OAT delivery, allowing chief narcologists to adopt independent decisions for their regions, though responses were distinct and often influenced implementation in other regions. Here, we describe the process by which many of the chief narcologists responded to the conflict in their respective regions at a specific point in time, with attention to how they dealt with their existing patients, internally displaced patients, and new patients seeking treatment due to disruptions in the illegal supply of opioids.

### Context

According to Ukraine's Public Health Center (PHC) within the Ministry of Health, HIV prevalence among the 346,000 PWID is 22.5% (8). At the beginning of the war, Ukraine had the largest OAT program in Eastern Europe and Central Asia with 17,232 patients officially enrolled at 233 government-funded sites. A parallel system of private clinics emerged starting in 2016 with changes in Order 200 with a minimum of 2,743 OAT patients at 18 privately-operated clinics, with an unknown number of OAT patients receiving treatment outside of officially recognized treatment programs (18). Many such patients could receive OAT as “prolonged detox” allowing these entrepreneurial private clinics to sell OAT, but not report to the CPH (only those patients maintained on OAT, rather than on prolonged detox that functionally operated as maintenance, were officially reported). Both private and entrepreneurial OAT clinics are mostly located in Eastern and Central Ukraine, use a fee-for-service model and are not necessarily included in the national OAT registry used for both tracking and medication procurement. Instead, private OAT clinics provide prescriptions that allow their patients to purchase OAT at commercial pharmacies that are licensed to store these medications (14). These two types of private clinics increased OAT by allowing people to avoid governmental registration and, in many cases, aside from notable exceptions (14), many of these clinics discontinued services early in the invasion. Furthermore, all OAT is manufactured in Ukraine as generic medications by two manufacturers in Kharkiv and Odesa that discontinued medication production because they were in conflict regions under attack by Russia; in July 2022, these manufacturers re-opened. Despite these shut-downs, there was sufficient medication supply in storage to treat over 21,000 patients continually through October 31, 2022 (n.b., there were 17,232 patients on OAT as of February 2022 leaving sufficient medication to enroll new patients though distribution efforts were disrupted in some regions).

The war-affected every aspect of OAT delivery and required clinicians to prioritize a diverse set of patients including (1) continuing existing governmental patients at their clinics; (2) continuing treatment for existing governmental patients from other regions (i.e., internally displaced patients); (3) continuing existing private patients from the same or other regions (i.e., both local and internally displaced patients); and (4) new patients who had not been on OAT but who opted for it during times of uncertainly due to elevated stress or disrupted supplies of illicit opioids. Though chief narcologists had been informed by the PHC that there was a sufficient supply of OAT nationally, they could not predict if the supply would be disrupted in the future with the uncertainties of the war and the continued changes in the conflict zones.

The number of patients seeking OAT at governmental sites increased as the country's curfew and the territorial defense patrols created shortages of illicit opioids and restricted illegal transactions of drugs. Moreover, OAT delivery became challenging with disruptions in transportation routes and increasing checkpoints as clinicians maintained clinical operations during active shelling, warning sirens, and limited options to seek safety, especially in the frontline areas. Joint efforts by governmental and non-governmental agencies partially restored OAT storage and delivery for clinics in government-controlled areas, but only for governmental clinics. These collaborative efforts were able to restore OAT supplies even to regions that had been occupied, as these areas continued to monitor and provide OAT for patients as they transited from east to west.

Because Ukraine was able to maintain governance during the war, the Ministry of Health emergently enacted Order 665 which allowed more flexible distribution of OAT, including to regions with the highest prevalence of internally displaced people, and Order 409, which allowed up to a 30-day supply of take-home dosing for patients. Though PHC informed chief narcologists about the availability of medication supplies, there was no specific guidance or recommendations on how to decide how to implement THD, how to make dose adjustments, or whether to prioritize enrollment of new or internally displaced patients. Consequently, each region evolved its strategy during weekly NIATx coaching calls, where stimulus lectures were provided to give examples from outside Ukraine and coaches supported narcologists to adopt practices to optimize patients with OUD, which we explored using qualitative interviews with leaders in the regions.

Since 2014, OAT scale-up has been guided using NIATx, a bundle of implementation tools that involve the customer, fix key problems, involves the chief narcologist who serves as the executive sponsor and change leader, uses ideas from outside the country by providing coaching and stimulus lectures from U.S. Collaborators, international travel to learn new ideas, and uses rapid cycle testing (19, 20). Four Ukrainian NIATx coaches are supervised by two United States. NIATx coaches that interact with the chief narcologists and his/her team weekly to guide incremental change projects, with results that have been used to guide health policy changes, respond to the introduction of the National Health System, COVID-19, and now, the war (12, 14).

#### Enrollment and procedures

**Figure 1:**
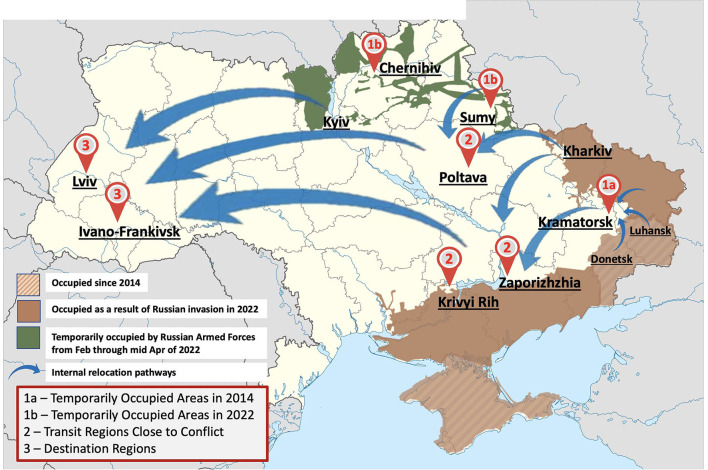
Interview site map relatively to the war situation in Ukraine as of April 15^th^, 2022.

Recognizing that regions of the country were impacted differently by the war, we categorized regions based on whether they were occupied by Russian forces, close to conflict with them, or were in regions without conflict but where internally displaced persons traveled. In each region, we also mapped OAT coverage to convey the extent to which chief narcologists had effectively scaled-up OAT (Figures 1A, B). Based on the severity of the military conflict, temporary occupation, and amount of internally displaced people, we classified regions in Ukraine as temporarily occupied (northern regions), transit regions proximal to conflict (central and southern regions), and destination regions remote from conflict (western regions). Members of our NIATx collaborative from each type of region were interviewed soon after the war started, from April 5, 2022, to April 22, 2022, for their perspectives and approach to addressing the evolving crisis in Ukraine and its impact on OAT delivery. These individuals had participated in weekly NIATx coaching calls. We included representative cities that we purposefully surveyed and represented in our map in Figure 1A, with additional details about the regions in terms of their OAT coverage and response during the war. A semi-structured interview guide was prepared that covered the way OAT delivery was provided before and during the war, at the time of the interview, and to gain insights into how different types of patients were prioritized for treatment and how services were delivered. Initially, the interview guide centered around the main research question: “How do you maintain the continuity of OAT at your site depending on the conflict-induced conditions in your region?” followed by “How did you adapt the OAT services to the most recent clinical guidelines issued after the Russian invasion?” Considering the ongoing OAT program scale-up and emerging new model of entrepreneurial private clinics, more questions were added focusing on the region-specific enrollment strategy for (1) new patients naïve to OAT; (2) patients on OAT at private sites who sought treatment; and (3) internally displaced patients on OAT who had arrived from conflict regions. According to the emerging insights of the conducted interviews, we made adaptations to the interview guide, including questions regarding people on active duty on OAT or seeking OAT, safety precautions put in place for their site, and strategies to address potential interruptions of OAT supply, etc. Each participant provided oral consent and interviews were recorded and transcribed and analyzed using thematic analysis. A summary of the strategies employed for OAT delivery is provided in Table 1.

**Table 1:** Regional descriptions and responses to OAT delivery.

**Characteristics**	**Temporarily occupied regions**			**Transit regions**			**Destination regions**	
	**Kramatorsk** **(Donetsk)**	**Sumy**	**Chernihiv**	**Krivyi rih** **(Dnipro)**	**Zaporizhzhia**	**Poltava**	**Lviv**	**Ivano-frankivsk**
OAT Coverage (2022)	Medium	Low	Medium	Low	Low	High	Medium	High
THD (%), Feb 1, 2022	73.6	85.5	75.5	91.0	89.3	80.4	80.8	94.5
THD (%), Apr 1, 2022	98.3	98.4	98.0	94.0	85.9	97.1	89.5	99.2
Dose Change, average (Feb to May 2022)								
Net Change in patients (Feb to May 2022)	−387	1	45	109	−151	152	389	53
Military zone	Yes	Yes	Yes	No	No	No	No	No
Temporary occupation	Yes	Yes	Yes	No	No	No	No	No
Saw closure of OAT sites	Yes	No	No	No	Yes	No	No	No
Lowered OAT dose	No	Yes	Yes	No	No	Yes	No	No
Used alternative OAT transportation Strategy	No	Yes (Delivered from Poltava)	No	No	No	Yes	No	No
Used 30-Day THD	Yes	Yes (rural patients)	Yes (rural patients)	No	No	No	No	No
Gave patients the option to purchase medication	No	No	No	No	No	Yes	Yes	No
Maintained a waiting list	No	No	No	No	Yes	Yes	No	No
Enrolled new patients from private clinics	No	No	No	No	No	No	Yes	Yes
Enrolled new patients, not previously on OAT	No	No	No	No	No	No	Yes	Yes

## Results

### Temporarily occupied regions

#### Prior experience with occupation in the east (Donetsk)

Though parts of Donetsk were occupied by Russia, and OAT banned since 2014, other parts of Donetsk like Kramatorsk, Slovyansk, and Mariupol had continued to provide OAT. Though the OAT sites in Mariupol closed early as the site was bombed, the chief narcologist from Kramatorsk compared her observations from 2014 with the present:

*From the last time we were occupied, there is one thing I learned very well: “If you see them coming, you should save your drug supplies as much as possible… if Russians establish control over the city, it will be impossible to negotiate. They will shoot down everything, and our patients will be captured with no treatment available to them”*.

Under such conditions, OAT providers prioritized the health of current patients by providing them with a 30-day THD supply to reduce individual risk to patients (and providers) while supporting the patients to have enough supply to move to safer regions. These clinicians readily accepted governmental patients transiting from nearby sites that had closed, did not reduce medication dosages for fear of withdrawal symptoms, but did not accept patients from private clinics or those who wanted to newly enroll on OAT. This medication conservation approach prioritized existing patients but was unable to address the needs of others who also needed OAT. This reduction in supply and high demand for OAT resulted in people being unable to access OAT, with reports of others who stalked current OAT patients and the clinic itself to obtain medications, at times causing harm to existing patients—another unintended consequence of the war.

The OAT clinic in Kramatorsk (Donetsk region), given its proximity to the conflict and perpetual response to air raid sirens from nearby bombings, limited its hours of operation to only a few hours in the morning 3 days per week. Nonetheless, staff spent considerable time hiding in basements not fully equipped as bomb shelters and at times, continued providing medications despite the risk. Some slept near the medication supply to protect it. Sites like Kramatorsk so close to the fighting planned for their patients to transfer elsewhere to safety by preparing a written treatment plan that could be conveyed to other sites.

#### No prior experience with occupation in the north (Sumy and Chernihiv)

Sumy and Chernihiv on the North border of Russia and/or Belarus were deeply impacted by shelling and military invasion and had to organize services to protect patients and staff alike, especially as OAT supplies became uncertain. Though all OAT clinics continued treatment uninterrupted in both regions, OAT delivery differed within the region. In general, nearly all patients were transitioned to 7–10 days of THD, except where there were active on-the-ground battles or for rural patients where they received a 30-day supply. One important change occurred as many OAT patients joined Ukraine's Territorial Defense Forces and asked their providers for the restriction of the number of days of THD to prevent them from losing medications on the battlefield. For clinics whose medication transportation routes were impeded, neighboring regions (e.g., Poltava) reallocated their supply to provide OAT. In such settings, doctors wanted to conserve medications and did not allow enrollment of new patients given the uncertainty of new supplies while others risked their lives by driving through enemy checkpoints to obtain needed medications. Providers recalled events in Crimea from 2014 when Russia banned all OAT and 10% of the OAT patients had died within 6 months from overdose or suicide (21). Once supply chains were re-established, however, new patients were prioritized for treatment.

### Transit regions close to conflict (Zaporizhzhia, Poltava, and Kryyv Rih)

Unlike Kramatorsk, which was quite near the fighting, larger cities in south-central parts of Ukraine became transitional zones for those fleeing the frontlines as patients on treatment would either relocate nearby for safety or saw such settings as temporary as they planned further migration westward. Thousands of people leaving the Donetsk and Luhansk regions traveled to the cities of Zaporizhzhia and Poltava early in the invasion. Poltava, with its central location, also received large numbers of internally displaced persons from Kharkiv and Sumy. These two nearby transitional settings provided treatment for all governmental program patients for 14 days if relocation was planned for a prolonged time. Those planning short-term transit were provided with a five-day medication supply to allow them to reach their next destination. In these 2 cities, narcologists struggled to decide how to best allocate medications given the uncertainty of their patients leaving, how many internally displaced patients would arrive and whether the war zone would expand and jeopardize their OAT supply chain. To manage these uncertainties, waiting lists were created that included both private clinics and new patients. Those from private clinics in Zaporizhzhia would be enrolled as existing patients transited to safer regions in the west. Despite being assured that there was sufficient OAT available by the PHC, their proximity to conflict limited their enrollment of new patients due to uncertainties in supply chain disruptions, despite considerable effort by Ukraine's PHC and non-governmental organizations to overcome obstacles. In Poltava, however, private patients were not directly transitioned into government clinics and instead, were treated similarly as new patients requiring full induction processes. Initially, it was recommended to existing patients to lower their OAT dosage to conserve medications and to allow enrollment of new patients, however, this recommendation was reversed when their medication supplies were assured when transportation logistics were not interrupted. Though there were no reports of people with OUD in the community accosting OAT patients as they left the clinic, they had heard of reports from the Donetsk region and secured support from the regional military authorities to provide protection.

Though Kryvyi Rih was close to the conflict, few internally displaced persons traveled there, perhaps because it is mostly isolated from transportation routes. Instead of conserving medications for such persons, they continued to focus on new patients who needed treatment, as they previously had successfully enrolled large numbers of new patients and simultaneously provided more extensive THD for up to 14 days. To reduce risk to clients, they provided THD for new patients (up to 2–3 days), which was a new strategy and promising practice to reduce demands on patients (and clinicians), as evidenced by the statement from the leading narcologist:

Before the war, we had 95% in take-home dosing, while the remaining 5%, those we didn't trust or those in the dose induction phase, would come daily. Since the air raid threat, after we got permission to dispense a 30-day supply, patients would get their methadone for two or even 3 days even during the induction phase if we felt they could be trusted.

### Destination regions in the far west (Lviv and Ivano-Frankivsk)

The largest number of internally displaced OAT patients traveled to Lviv, near the Polish border, and Ivano-Frankivsk, in the southwest. These internally displaced persons included PWID not on treatment, PWID from private clinics in the conflict zones, and governmental clinic patients. Most of these were men, who account for most PWID and, if between 18–60 years old, could not leave Ukraine and were eligible for conscription. These 2 regions, before the war, had relatively low numbers of PWID yet a relatively high proportion of patients on OAT. Though these regions were experienced with efficiently enrolling patients, they had slightly different responses during the war. Lviv, which received the overwhelming majority of internally displaced persons, effectively scaled up treatment, but without relaxing THD policies. Unlike Ivano-Frankivsk, which liberally enrolled new patients or those from private clinics, Lviv did so as well but adopted a medication conservation policy. This drug conservation policy emerged due to the sheer number of new patients (*N* = 389 new patients) trying to enroll balanced with concerns about an insufficient supply of medications. To ensure that there would be sufficient medication supply, narcologists in Lviv asked patients to voluntarily “purchase” a 10-day supply from pharmacies to allow their government supply to last longer—all patients agreed as a communal practice. At both sites, there were no restrictions placed on enrolling new patients, including internally displaced persons who traveled west and whose supply of illegal opioids had been disrupted. As these sites had virtually no clinical information about patients from private clinics, governmental clinics in Lviv imposed stringent requirements to enter inpatient treatment for an initial day for diagnosis and screening (including for HIV, HCV, and tuberculosis testing) activities. Of interest and unlike clinics in Poltava, they started private patients on induction courses of medications, but when they observed symptoms of opioid withdrawal, they accelerated their dosage escalation to achieve a clinically responsive dose. Of note, they reported that some patients from private clinics reported being on dosages over 300 mg per day, a dosage not supported by clinical guidelines, which they were not willing to restart without clinical observation:

“*There was also another problem. For instance, in Kharkiv, at three different private sites, one patient had been prescribed 300 mg of methadone daily… It was a shock for us! We slowly “brought them to mind?”, and they become more like our local patients. Of course, we started comparing them to our polite and tidy patients, but those (i.e., internally displaced patients) who came here are different. They were demanding high doses. Eventually, somehow they understood and became calmer and “normal?”*.

## Discussion

In 2014, with Russia's first invasion of Ukraine since regaining its independence in 1991, Ukraine experienced an unprecedented humanitarian crisis (3). Even the regional conflict affected the struggling post-soviet health care system by damaging facilities in Donbas and causing internal relocation of people from the invaded regions after the annexation of Crimea (3). The war escalation in February 2022 resulted in one-third of Ukraine's 45 million people becoming displaced, either internally or externally as refugees. While several other studies discuss the challenges of healthcare provision for displaced people during the armed conflicts with the violation of international humanitarian laws in Ukraine and elsewhere (3, 22, 23), there are no documented responses to conflict for vulnerable populations like PWID. The context is especially crucial as the invading forces from Russia are especially hostile toward OAT, an evidence-based treatment for OUD, and ban it entirely within Russia and now in occupied regions of Ukraine (21, 24). Findings here provide new and important insights into how to respond to a public health emergency, and future preparedness strategies, from an implementation science perspective as the participants were exposed to collaborative learning interventions to respond to the dynamic nature of the war, using NIATx in particular (12).

Though each of the narcologists interviewed was part of a NIATx collaborative, there were some overlapping responses to OAT policies based on their proximity to and prior experience with the war, yet some of their responses were unique and represent promising practices that can lead to future innovation. As part of collaborative learning, OAT experts were able to share their experiences and learn from each other. While it was emphasized during collaborative NIATx meetings that there was a sufficient supply of OAT in the country and more OAT forthcoming through support from PHC, some adopted a medication conservation strategy as the processes of war are dynamic and uncertain. Indeed, after over 100 days of the war, regions in the North and near the capital Kyiv have become more stable and returned to pre-war operations, while those in the east and south worsen.

Central to the response during the war is the need to ensure continued treatment for those already prescribed OAT (including those at private clinics), but also to use this as an opportunity to stabilize individuals who are actively injecting opioids and who have encountered a meaningful moment when treatment may be markedly better than continued injecting as supplies of illegal opioids become limited. Important in this process is the importance of ensuring treatment access to all patients on OAT, irrespective of whether they are treated in governmental or private clinics. Certainly, for those in governmental clinics, there is a national database (SyRex) where all OAT patients, past or present (25), are recorded, and information can be reasonably well-accessed. For private patients, however, such information systems are limited or completely unavailable, potentially suggesting the need for a national reporting system, much like the prescription drug monitoring program (PDMP) in the United States. At a minimum, clinicians anywhere in the country could access patient information using a PDMP to verify the prescription of OAT along with verifying the recent dose.

One of the potential unintended consequences of war and interruptions of OAT is excessive overdose or suicide by those either discontinuing or seeking OAT and outbreaks of HIV and HCV—which was observed when Crimea was illegally annexed by Russia and all OAT was banned (26). It is especially concerning that in a country where HIV prevalence is so high among PWID (22.5%), not only will many OAT patients inject when their dose is reduced, but when risky sharing of injecting equipment will occur as injection frequency increases during stress and sterile syringes become scarce. For those patients with HIV on OAT, treatment disruptions also possess concerns about discontinuation from antiretroviral therapy (9, 27), which would fuel excessive mortality and potentially increase transmission.

Of importance here, however, is that while 858 of the 17,232 patients on OAT dropped from treatment during the first month, the country has rebounded with 19,342 patients on OAT as of August 1, 2022 (12), and accelerated its scale-up to pre-war levels aside from regions that are partially occupied. This represents resilience and commitment by governmental and non-governmental agencies who prioritize vulnerable populations alongside the OAT providers throughout the country to continue to participate in NIATx collaborative learning activities. OAT providers out of necessity have introduced some promising practices as observed from their interviews, including THD during induction, more expanded THD policies for patients newly starting OAT, and, in some cases, rapidly responding to the need for patients to continue treatment from private clinics. Moreover, they have learned to rapidly respond clinically to internally displaced persons from private clinics and provided accelerated induction procedures to meet patient needs. Specifically, there was the impression that patients from the East were “different” and did not adhere to the norms of their clinics. Nonetheless, these sites overcame these differences and focused on continuity of care and enrolling new patients despite these perceptions. No matter how atrocious the experiences with war are, there have emerged innovations to treatment, much like those observed during the COVID-19 pandemic, where narcologists rapidly responded to relaxation in governmental policies during the war by shifting to THD and reducing demands on patients and clinicians (13). They did this through their collaborative learning, meanwhile succeeding in enrolling new patients more efficiently but doing so with significantly higher retention levels and lower mortality (28).

## Conclusions

There are no international guidelines available to address healthcare responses during the war for vulnerable populations, specifically for people with OUD who have or are at high risk for HIV. The findings here provide an overview for a response depending on proximity to conflict zones. Key in this response, however, has been the ability to engage in ongoing collaborative learning and maintain a meaningful supply of medications despite intermittent interruptions. In the absence of such a response, there is the risk of unintended psychological distress and heightened mortality, alongside outbreaks of HIV and HCV, which would undermine Ukraine's progressive public health response thus far.

## Data availability statement

The original contributions presented in the study are included in the article/supplementary material, further inquiries can be directed to the corresponding author.

## Ethics statement

The studies involving human participants were reviewed and approved by Yale Human Investigation Committee. The patients/participants provided their written informed consent to participate in this study. Written informed consent was obtained from the individual(s) for the publication of any potentially identifiable images or data included in this article.

## Author contributions

RI: conceptualization, data collection, methodology, and writing—original draft. SG: data curation, writing—original draft, and methodology. AM and DB: conceptualization and data collection. LM and SF: conceptualization. IP: conceptualization, data curation, and methodology. TF: data collection. ZI: methodology. FLA: conceptualization, methodology, writing—original draft, and writing—review editing. All authors contributed to the article and approved the submitted version.
